# A Monocentric Retrospective Study of AUC/MIC Ratio of Vancomycin Associated with Clinical Outcomes and Nephrotoxicity in Patients with Enterococcal Infections

**DOI:** 10.3390/pharmaceutics13091378

**Published:** 2021-08-31

**Authors:** Wasan Katip, Peninnah Oberdorfer

**Affiliations:** 1 Department of Pharmaceutical Care, Faculty of Pharmacy, Chiang Mai University, Chiang Mai 50200, Thailand; 2 Epidemiology Research Group of Infectious Disease (ERGID), Chiang Mai University, Chiang Mai 50200, Thailand; aoberdor@med.cmu.ac.th; 3 Division of Infectious Diseases, Department of Pediatrics, Faculty of Medicine, Chiang Mai University, Chiang Mai 50200, Thailand

**Keywords:** therapeutic drug monitoring, vancomycin, enterococcal infections, AUC/MIC

## Abstract

Vancomycin is an antibiotic commonly used for the treatment of enterococcal infections. However, there is no clear correlation regarding of vancomycin area under the curve/minimum inhibitory concentration (AUC/MIC) ratio and clinical outcomes for the treatment of enterococcal infections. The aims of this study were to evaluate the relationship of vancomycin AUC/MIC ratio in patients with clinical outcomes and nephrotoxicity for patients with documented enterococcal infections. A Bayesian technique was used to calculate the average vancomycin AUC_0–24_. The MIC was determined using the VITEK 2 automated microbiology system, and the average AUC_0–24_/MIC value was calculated for the first 72 h of therapy. All medical records of patients prescribed vancomycin with therapeutic drug monitoring were collected during January 2010–October 2020 at Chiang Mai University Hospital (CMUH). A retrospective single-center cohort of 312 participants were met the inclusion criteria. The results of this study showed that, a vancomycin AUC/MIC of ≥400 mg·h/L was associated with significant differences in clinical response compared to a vancomycin AUC/MIC of <400 mg·h/L (aHR: 0.50, 95% CI: 0.26–0.97; *p* = 0.042). Likewise, a vancomycin AUC/MIC of ≥400 mg·h/L was associated with significant differences in the microbiological response (aHR: 0.37, 95% CI: 0.14–0.94; *p* = 0.036), compared to a vancomycin AUC/MIC of <400 mg·h/L. However, nephrotoxicity in patients with a vancomycin AUC/MIC of ≥400 mg·h/L was higher than those with a vancomycin AUC/MIC of <400 mg·h/L (aHR: 3.96, 95% CI: 1.09–14.47; *p* = 0.037). Declining renal function may be a result of high vancomycin concentrations. In addition, declining renal function (e.g., failure to resolve the focus of infection, co-administration of other antibiotics) might result in higher AUC/MIC. We found a target vancomycin AUC/MIC of ≥400 mg·h/L and this AUC/MIC target value could be optimal for the use for monitoring treatment of enterococcal infections. Thus, vancomycin dosage must be adjusted to achieve the AUC/MIC target and closely monitored for renal function. These findings are not transferable to critically ill patients.

## 1. Introduction

*Enterococci* are Gram-positive facultative anaerobic cocci in short and medium chains that produce complicated infections. They are naturally found in gastrointestinal colonizers, but they can also be a cause of urinary tract infections (UTIs), bacteremia, pelvic infections, wound infections, and infective endocarditis, as well as intra-abdominal infections and meningitis in rare cases [1]. These can range from simple infections to life-threatening sepsis, which is a serious problem around the world [1]. The prevalence of penicillin resistant *Enterococcus* spp. at Chiang Mai University Hospital (CMUH) from January 2010–December 2020 also increased from 17.00% to 43.51%, respectively [2]. Vancomycin is a glycopeptide antibiotic that is commonly used to treat infections caused by methicillin-resistant *Staphylococcus aureus* (MRSA), methicillin-resistant *Staphylococcus epidermidis* (MRSE), *Enterococcus faecalis*, and *Enterococcus faecium*. Vancomycin exhibits a concentration-independent reaction [3,4]. Furthermore, the area under the curve of the vancomycin concentration-time graph above the minimum inhibitory concentration (AUC/MIC) is the pharmacokinetic (PK)/pharmacodynamic (PD) parameter that best correlates with favorable outcomes of invasive MRSA infections [5,6].

Despite the fact that there are significant gaps in our understanding of the therapeutic use and effectiveness of vancomycin in patients with enterococcal infection, it is nevertheless utilized in these patients [4,7,8]. Furthermore, there is no apparent link between vancomycin AUC/MIC ratio and favorable patient outcomes in enterococcal infection [9,10]. Furthermore, increasing the vancomycin AUC/MIC ratio may raise the likelihood of negative effects such as nephrotoxicity. While a newly published consensus paper mentions an elevated risk of nephrotoxicity, the authors emphasize that there is limited data available [11,12]. The objectives of this study were to evaluate if vancomycin AUC/MIC ratios are associated to clinical and microbiological results, as well as vancomycin AUC/MIC ratio-related nephrotoxicity in patients with enterococcal infections.

## 2. Materials and Methods

### 2.1. Methods

A retrospective study of all in-patients treated with intravenous vancomycin at CMUH, a university-affiliated hospital in Chiang Mai, Thailand, between January 2010–October 2020, was performed. The Chiang Mai University (CMU) Faculty of Medicine’s ethics committee on human research (NONE-2563-07835) approved this study with a waiver of informed consent for retrospective data collection on the condition that data be stored anonymously. According to the assessments of infectious disease (ID) physicians, the criteria used to identify and classify infections were obtained from the Centers for Disease Control and Prevention (CDC) [13].

The following criteria were used to determine eligibility: (i) age over 18 years; (ii) treatment with vancomycin for more than three days in patients with microbiological confirmation of infection with *Enterococci* spp. resistant to penicillin but susceptible to vancomycin (for patients with more than one episode of enterococcal infection, only first episode during the study period was included); and (iii) at least one measure resistant to penicillin but susceptible to vancomycin. Patients were excluded from the analysis if they were (i) previously exposed to intravenous vancomycin within seven days; (ii) pregnant; (iii) undergoing hemodialysis; or had (iv) polymicrobial infection or infection with isolates resistant to vancomycin; (v) *Enterococcus* cultures assessed as colonizers or contaminants; or (vi) incomplete patient records.

Vancomycin was administered as a 60-min intravenous infusion (in patients with normal renal function, 1 g every 12 h). In the instance of renal impairment, the doses were adjusted according to the CMUH guide protocol’s recommendations. The pharmacy and infectious disease physicians (The Pharmacy and Therapeutics Committee) developed the CMUH protocol, which is documented in the CMU antibiotic guidebook and recommended for usage in the hospital.

The vancomycin dose was adjusted based on renal function in individuals with renal failure (creatinine clearance; CL_CR_ > 50 mL/min: 1 g every 12 h, CL_CR_ 40–49: 750 mg every 12 h, CL_CR_ 40 mL/min: 750 g every 48–120 h).

### 2.2. Data Collection

Data was collected from patient medical records and computerized hospital systems. Age, gender, body weight, height, vancomycin dose, duration of treatment, underlying disease (cerebrovascular disease, solid tumor, hematologic malignancy, chronic kidney disease, chronic liver disease, coronary artery disease, or diabetes mellitus), length of hospital stay, acute physiology and chronic health evaluation (APACHE II) score, glomerular filtration rate (GFR), baseline serum creatinine, types of nephrotoxic medications, source of enterococcal infection, enterococcal species, mortality status, and bacteriological data were included in the collection. The vancomycin concentration measured shortly before the next dose was used to determine the trough levels. As recommended by the guidelines [4,7], the initial vancomycin trough concentration was determined at steady-state (before the fourth dose of vancomycin administration). Serum must be obtained within 30 min of the following dose to accurately assess the trough level [14].

### 2.3. Pharmacokinetic Data for Vancomycin

The vancomycin dose administration dates and times, as well as the blood draw for vancomycin concentrations, were noted and submitted into the BestDose software (Saban Research Institute, Children’s Hospital Los Angeles, Los Angeles, CA, USA).

The Bayesian technique was used to calculate the vancomycin AUC over a 24-h period (AUC_24_). BestDose software version 1.3.041 was used to calculate individual pharmacokinetic parameters for each patient [15]. This program was used to personalize the dose of vancomycin in patients [16].

Bayesian dose optimization software calculates a Bayesian posterior parameter value distribution for the patient, using well-developed vancomycin population pharmacokinetics for adults as the Bayesian prior and the individual patient’s observed drug concentrations in the data file [17]. BestDose uses nonparametric statistics to indicate, for example, that the Bayesian preliminary consists of discrete “support points” of which each are a collection of parameter values (e.g., distribution volume) and an associated probability for the parameter set. The probabilities for these support points are recalculated as the joint probability distribution for that patient for the “Bayesian posterior”, according to the patient’s data [18]. In fact, the patient was updated in the population model (Bayesian previous). The AUC was taken in 72 h (AUC_72_) from BestDose, which used the algorithms for trapezoidal approximation on the concentrations of the median Bayesian post-parameter values, as well as the patient’s dosing and covariate data, calculated approximately every hour. The AUC_72_ treatment was evaluated, as it is important to achieve the target early, especially in bacteremia cases [19]. By dividing the AUC_72_ by 3, the average AUC_24_ was calculated. The automated microbiology system VITEK 2 MIC method was used to calculate the AUC_24_/MIC.

### 2.4. Statistical Analyses

The study sample was separated into two groups for statistical analysis: patients with a vancomycin AUC/MIC of <400 mg·h/L (the control group) and patients with a vancomycin AUC/MIC of ≥400 mg·h/L (the experimental group). The student’s *t*-test was used to compare continuous variables expressed as means and standard deviations. Categorical variables were reported as frequencies and percentages, and Fisher’s exact test was used to compare them as necessary.

The primary outcomes were clinical and microbiological outcomes, as well as nephrotoxicity. To define independent variables related to the primary endpoint, univariate variables that were associated with clinical and microbiological results, as well as nephrotoxicity (*p* < 0.20), were included in a nominal logistic multivariate analysis (together with the vancomycin AUC/MIC group). In addition, all variables that revealed a trend toward association with the outcomes were forced into the model. A Cox proportional hazards model was also used to explain the time to clinical and microbiological outcomes. Stata software, version 14 (Stata-Corp, College Station, TX, USA) was used for data analysis and interpretation. *p*-values of <0.05 with two tails were considered statistically significant.

### 2.5. Outcome Measurement

The primary endpoint was clinical response to therapy, which was evaluated at the end of vancomycin treatment for enterococcal infection while on the drug. At the completion of treatment, clinical response was measured by the resolution or partial resolution of fever, leukocytosis, and local signs and symptoms of enterococcal infections. The CDC provided the criteria for identifying and classifying the infections [13]. Failure to achieve all clinical response criteria was characterized as a clinical failure. After the initial positive culture from the infection site, microbiological response was defined as two consecutive Enterococcus-negative cultures of clinical samples collected at the end of therapy, whereas microbiological failure was defined as Enterococcus persistence in subsequent cultures of the specimen due to the initial causative organism. A rise in serum creatinine (SCr) > 0.5 mg/dL above baseline, or a 50% increase from baseline in consecutive daily readings and a decrease in calculated creatinine CLc_R_ of 50% from baseline on two consecutive days in the absence of an alternative explanation were classified as nephrotoxicity [8].

### 2.6. Antimicrobial Susceptibility Test

At CMUH’s division of Clinical Microbiology, all isolates were identified as *Enterococcus* spp. using conventional methods. If the microorganisms met the Clinical and Laboratory Standards Institute’s standards (MIC of 8 g/mL for ampicillin and 4 g/mL for vancomycin), they were classified sensitive to ampicillin or vancomycin [20]. The VITEK 2 (bioMerieux, Inc., Marcy I ‘Etoile, France) automated microbiology system was used in the clinical laboratory to perform the species detection and susceptibility tests.

## 3. Results

**Figure 1:**
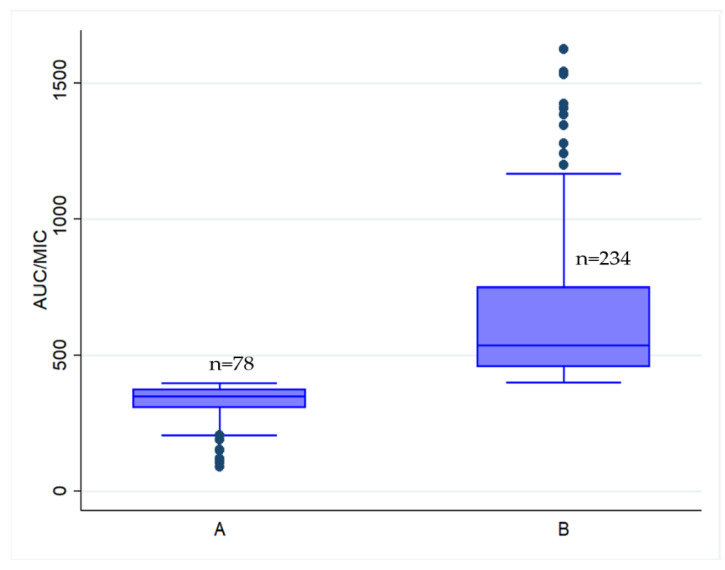
Distribution of AUC/MIC values. (**A**) AUC/MIC of <400 mg·h/L and (**B**) AUC/MIC of ≥400 mg·h/L.

During this study, 312 participants who were hospitalized with enterococcal infections and met the inclusion criteria were evaluated. One hundred and seventy-six cases (56.41%) were female, with a mean age of 61.16 ± 18.15 years. The most common underlying illnesses were coronary artery disease and chronic renal disease (Table 1). Ampicillin resistance was found in all 312 Enterococcus species, although vancomycin susceptibility was found in all. The vancomycin MIC of Enterococcus species strains was 0.99 ± 0.44 μg/mL (mean ± SD). Seventy-four patients (23.72%) were in the vancomycin AUC/MIC < 400 mg·h/L group and 238 patients (76.28%) were in the vancomycin AUC/MIC ≥ 400 mg·h/L group. The comparisons of patient characteristics between the two AUC/MIC groups are shown in Table 1, exhibiting that their characteristics are identical. Figure 1 shows the distribution of the AUC/MIC values between the groups of AUC/MIC < 400 and ≥400 mg·h/L.

BestDose program uses a Bayesian estimation algorithm to update preset pharmacokinetic parameters (prior probabilities) with the available data. The prior distribution of parameters derived from BestDose software (Table 2).

The overall rate of clinical failure was 17.31%, seen in 22.97% and 15.55% of patients in the vancomycin AUC/MIC < 400 mg·h/L and vancomycin AUC/MIC ≥ 400 mg·h/L groups, respectively. The rate of microbiological failure observed was 8.01%, seen in 12.16% and 6.72% of the patients in the vancomycin AUC/MIC < 400 mg·h/L and vancomycin AUC/MIC ≥ 400 mg·h/L groups, respectively. However, the rate of nephrotoxicity observed was 12.18%, seen in 3.85% and 14.96% of the patients in the vancomycin AUC/MIC < 400 mg·h/L and vancomycin AUC/MIC ≥ 400 mg·h/L groups, respectively (Table 3).

The multivariate Cox regression analysis showed that a vancomycin AUC/MIC ≥ 400 mg·h/L was associated with a significant decrease in clinical failure (aHR: 0.50, 95% CI: 0.26–0.97; *p* = 0.042), and microbiological failure (aHR: 0.37, 95% CI: 0.14–0.94; *p* = 0.036). However, a vancomycin AUC/MIC of ≥400 mg·h/L substantially increased the risk of nephrotoxicity (aHR: 3.96, 95% CI: 1.09–14.47; *p* = 0.037), compared to a vancomycin AUC/MIC of <400 mg·h/L (Table 3).

## 4. Discussion

A vancomycin AUC/MIC of ≥400 mg·h/L was associated with lower clinical and microbiological failure in patients with enterococcal infections than a vancomycin AUC/MIC of <400 mg·h/L. By contrast, higher rates of nephrotoxicity were related to a vancomycin AUC/MIC of <400 mg·h/L. A recent clinical study in patients with enterococcal infections exhibited the effectiveness of a vancomycin AUC/MIC of ≥400 mg·h/L. Dose adjustment to achieve a vancomycin AUC/MIC of ≥400 mg·h/L is recommended to achieve a favorable outcome. However, an AUC/MIC of ≥400 mg·h/L was associated with an increased rate of nephrotoxicity. Therefore, renal function should be closely monitored.

For decades, vancomycin, a glycopeptide antibiotic, has been the first-line treatment for MRSA infections, as well as *enterococci* resistant to ampicillin. Vancomycin has concentration-independent (time-dependent) effects, modest bactericidal activity, and a short post-antibiotic effect (PAE) in vitro [21]. Vancomycin caused shorter PAEs on *E. faecalis* (0.5–1.0 h) than *S. aureus* (1.3–1.8 h) at equal doses [22]. In order to enhance the efficacy of vancomycin against MRSA infection and reduce its toxicity in patients, serum vancomycin levels must be monitored in clinical practice [4,8]. Vancomycin should have an area under the curve AUC/MIC ratio of 400–600 mg·h/L [8]. The American Society of Health-System Pharmacists (ASHP), Infectious Diseases Society of America (IDSA), Pediatric Infectious Diseases Society (PIDS), and Society of Infectious Diseases (SID) recently recommended using Bayesian software programs to design vancomycin dosing regimens [10]. Bayesian-guided dosing was supported by Bayesian theorem. Patients’ pharmacokinetic parameter values and individual drug concentration data were used to recalculate population pharmacokinetic variables, such as the volume of distribution (Vd) or drug clearance (CL) of prior values (Bayesian prior) [10].

AUC/MIC is the ideal method for evaluating vancomycin efficacy and ensuring appropriate PD exposure in adults, based on animal models, in vitro experiments, and limited human trials [10]. Moise-Broder et al. [5] investigated at the use of AUC/MIC in a group of adult patients with *S. aureus* pneumonia and discovered that a vancomycin AUC/MIC ≥ 400 mg·h/L was the best predictor of clinical and bacteriologic success. Thus, in the treatment of *S. aureus* infections, PK/PD targets have been identified. However, there are few data to validate such goals in enterococcal infection and no agreement on the best PK/PD parameters for treating enterococcal infection [4,7,8]. Furthermore, the PK/PD studies that have demonstrated that a greater AUC/MIC is associated with favorable outcomes from enterococcal infection are still debated [9,10].

In response, this study contributed to the existing data on the correlation between vancomycin AUC/MIC and clinical outcomes and nephrotoxicity, addressing the concerns of several of the authors of the agreement review.

In our study, we observed that a vancomycin AUC/MIC of ≥400 mg·h/L was associated with lower clinical failure rate (aHR: 0.50, 95% CI: 0.26–0.97; *p* = 0.042), and lower microbiological failure rate (aHR: 2.46, 95% CI: 1.11–5.47; *p* = 0.027), compared to a vancomycin AUC/MIC of <400 mg·h/L.

A study with findings similar to the present analysis was a retrospective, single-center observational study at Tan Tock Seng Hospital (TTSH) of enterococcal bacteremia treated with vancomycin between 1 January 2009 and 31 May 2015 in Singapore that published in 2018 [9]. The primary outcome of this study was a 30-day all-cause mortality. The vancomycin AUC_0–24_/MIC value related to 30-day mortality was identified using classification and regression tree analysis (CART). E-test was utilized by the researchers to determine MICs. The average vancomycin AUC_24_ was calculated using a Bayesian technique. This study found that failure to achieve a CART-derived vancomycin AUC/MIC_Etest_ value of ≥389 mg·h/L was independently associated with 30-day mortality (odds ratio, 6.83 [95% CI, 1.51–30.84]; *p* = 0.01) and was associated with lower mortality (*p* = 0.017). A vancomycin AUC/MIC_Etest_ value of 389 obtained within 72 h of vancomycin medication was associated with lower mortality in patients with *E. faecium* bacteremia [9].

In contrast, a retrospective study by Nakakura et al. [10] found no significant difference in mortality between the proportion of patients with vancomycin AUC_24_/MIC < 389 and ≥389. Furthermore, in individuals with *E. faecium* bacteraemia, the AUC_24_/MIC ratio was not related to mortality in these patients, however, the severity of the condition was related to mortality [10]. Because this was a retrospective study with a small sample size, it may have revealed unjustified correlations. Furthermore, the mortality rates between the groups in this study were compared using a crude comparison method that did not adjust for confounding variables [10].

Nephrotoxicity is typical in vancomycin-treated patients, with the most serious side effects described in the present literature [4,8]. According to a patient survey and the degree of renal failure [7,8], the prevalence of vancomycin-related acute kidney damage (AKI) ranges from 5–43% [8]. A vancomycin AUC threshold for nephrotoxicity is still controversial. However, an increased vancomycin AUC was found to significantly increase nephrotoxicity. Suzuki et al. [12] examined the relationship between vancomycin AUC and AKI. AUC values of 600–800 mg·h/L were found in the majority of patients who had AKI, compared to 400–600 mg·h/L in those who did not develop AKI (*p* = 0.014).

In our study, a high AUC/MIC ratio (≥400 mg·h/L) was also associated with a high rate of nephrotoxicity (aHR: 3.96, 95% CI: 1.09–14.47; *p* = 0.037), when compared to a low AUC/MIC ratio (<400 mg·h/L). One of the risk factors related to vancomycin-induced nephrotoxicity was renal impairment (aHR: 3.27, 95% CI: 1.92–5.56; *p* = 0.03) [23]. In our study, there were basal differences in Scr and GFR between patients. Although those differences were not statistically significant (Table 1), they could be responsible for a worsening of the renal function. This difference, however, was found in most retrospective studies and was difficult to make them equal for both groups. In this study, therefore, we used multivariate Cox proportional hazards model to adjust the difference between the vancomycin AUC/MIC ≥400 mg·h/L and vancomycin AUC/MIC <400 mg·h/L groups. With this statistical analysis, baseline Scr and GFR as well as other baseline values were adjusted to be the same in both groups. The median (range) time from initiation of vancomycin therapy to meet nephrotoxicity criteria was eight (three to 17) days. However, the majority (80%) of patients experienced recovery of their Scr and GFR within 12 days after vancomycin was discontinued.

This is consistent with Neely et al. [24], who conducted a prospective research in which 252 individuals were followed using troughs of 10–20 mg/L in year 1 vs. Bayesian-estimated AUC values of 400 mg·h/L in years 2 and 3. Nephrotoxicity occurred in 8% of patients in the first year and 0% and 2% of subjects in the second and third years, respectively (*p* = 0.01). The average trough level and AUC related with AKI were 15.7 mg/L and 625 mg·h/L, respectively, compared to 8.7 mg/L and 423 mg·h/L in non-AKI patients (*p* = 0.02). When compared to trough concentration targets, AUC-guided, Bayesian estimation-assisted vancomycin dosing was associated with less nephrotoxicity, less per-patient blood sampling, and a shorter treatment time without affecting efficacy [24].

In contrast, a single-center, retrospective, pre-post quasi-experimental study [25] examined the incidence of AKI before and after a health-system-wide switch from trough-guided to AUC-guided therapy. AKI was the primary endpoint in participants who were administered vancomycin with or without piperacillin-tazobactam (P-T). In both the trough-guided and AUC-guided groups, the incidence of AKI was significantly greater in patients who received concomitant P-T, compared to those who did not receive concomitant P-T (17.8% vs. 7.4%; *p* = 0.003) in the AUC-guided group (13.6% vs. 3.8%; *p* = 0.0011). Between trough- and AUC-guided vancomycin dosage, the incidence of AKI did not differ significantly. Regardless the dosing strategy, caution is required while combining vancomycin and P-T [25].

Moreover, Zasowski et al. [26] reported a multicenter, retrospective investigation of vancomycin exposure–toxicity thresholds in 2017. The patients in this study were given vancomycin from 2014–2015, and nephrotoxicity was defined the same way as in the Australian study. There were 323 patients in total, with 20 of them (6.2%) suffering nephrotoxicity. Nephrotoxicity was dramatically higher among patients with an AUC_24_ of 677 mg·h/L, according to a classification and regression tree (CART) analysis. Positive predictive values were low for all ranges studied, but the AUC_24_ of 677 mg·h/L had a negative predictive value of 96.7% (adjusted OR, 3.73; 95% CI, 1.646–8.470) [25].

Several reasons might explain the significant differences in the nephrotoxicity rates. Firstly, the definition of AKI differs between prior studies and our study. Most studies define AKI as a 0.3 or 50% increase in SCr from baseline, however our study classified AKI as a 0.5 or 50% increase in SCr from baseline and a decrease in calculated CLc_R_ of 50% from baseline on two consecutive days. Secondly, most studies have used AUC, whereas our study used AUC/MIC. There are increased reports of vancomycin-induced nephrotoxicity as a result of the increased interest in targeting to ensure a higher possibility of reaching an AUC/MIC of ≥400 in the presence of higher MICs. Thirdly, the use of vancomycin in combination with P-T or a nephrotoxic agent was found to be associated with greater nephrotoxicity.

The distribution of AUC/MIC in groups A and B (Figure 1) show a broad distribution of AUC/MIC values with a certain number of patients attaining extremely high values. Consequently, this is a main determinant of nephrotoxicity. Thus, vancomycin administered by continuous infusion (CI) can resolve the wide distribution of AUC/MIC values. This is supported by Cristallini et al. [27] in a prospective pharmacokinetic study was conducted to evaluate a new vancomycin dosage regimen given via CI to patients with sepsis. Between January 2012–May 2013, all adult patients with sepsis admitted to a mixed intensive care unit (ICU) were treated with a new vancomycin CI regimen that included a loading dose of 35 mg/kg of body weight given as a 4 h infusion, followed by a daily CI dose adapted to CL_CR_, as approximated by the Cockcroft-Gault formula (median dose, 2112 [1500–2838] mg). For several MICs, vancomycin concentrations were used to predict AUC_0–24_/MIC ratios of ≥400 mg·h/L. Target serum vancomycin concentrations of 15 mg/L at T3 for a MIC of 1.0 mg/L resulted in AUC_0–24_/MIC ratios of ≥400 mg·h/L in all patients. Most patients were able to achieve their goal serum concentrations shortly with this vancomycin regimen.

In a large retrospective multivariate analysis of 1430 patients who received vancomycin in a tertiary hospital, Hanrahan et al. [28] found that intermittent infusion (II) was associated with a significantly higher risk of nephrotoxicity than CI (OR = 8.204; *p*= 0.001). Hanrahan et al. [29] also published an updated meta-analysis that included all publications comparing nephrotoxicity in CI and II. The key words ‘vancomycin’ and ‘continuous’ or ‘intermittent’ or ‘infusion’ or ‘discontinuous’ or ‘administration’ were searched in the PubMed, EMBASE, and Cochrane Reviews databases. In the final analysis, seven studies were included in the study. A non-significant trend of lower nephrotoxicity in those who received vancomycin via CI (risk ratio = 0.799, 95% CI 0.523–1.220; *p* = 0.299) was found using a random-effects model. In addition to recent articles supporting CI, this meta-analysis demonstrated that CI should be considered the preferred delivery strategy for vancomycin to lower the risk for nephrotoxicity [29]. However, larger prospective random controlled trials will need to verify these findings in the future.

The dosage of vancomycin administration to achieve AUC_24_/MIC ratios of ≥400 mg·h/L was determined. Based on our data of the baseline characteristics of patients with enterococcal infection treated with vancomycin (Table 1.), a mean vancomycin dose of 28.4 ± 5.2 mg/kg correlated with an AUC_24_/MIC of ≥400 mg·h/L. In addition, based on previous data of our study [30], a loading dose of 30 mg/kg vancomycin and subsequent dose of 20 mg/kg every 8 h to achieve AUC_24_/MIC ≥400 mg·h/L on the first day of therapy were recommended. Moreover, for achieving AUC_24_/MIC ratios of ≥400 mg·h/L, a new vancomycin CI regimen consisting of a loading dose of 35 mg/kg of body weight given as a 4-h infusion followed by a daily CI dose (median dose: 2112 [1500–2838] mg), can be implemented [27]. Therefore, for initial achievement of PK/PD targets for better clinical outcomes, vancomycin should be used at a loading dose of 30–35 mg/kg to cover enterococcal infection. Finally, therapeutic drug monitoring is required for further dosage adjustments to achieve AUC_24_/MIC ratios of ≥400 mg·h/L.

*E. faecalis* and *E. faecium* are the most prevalent enterococcal health-care-associated infection isolates, and their ability to survive heat, UV radiation, and aseptic solutions [31] permits them to remain in the hospital ecosystem [31]. If healthcare professionals do not wash their hands, *Enterococcus* can spread. *E. faecalis* can also be found on improperly cleaned catheters, dialysis ports, and other medical devices. As a result, before and after any manipulation of the catheter site or device, thorough handwashing with soap and water is required [31,32,33]. *Enterococcus* is also a common cause of catheter-associated urinary tract infections (CA-UTI) and catheter-associated bloodstream infections (CA-BSI) [31,32]. CA-UTI are caused by enterococcal colonization of the external catheter surface, which usually results in the formation of a biofilm [31,32,33]. Biofilm formation has also been seen more frequently in *E. faecalis* [34] and *E. faecium* [35] device-related BSI. As a result, it has been proposed to be a virulence marker. Biofilms provide a protective environment for organisms that are poorly penetrated by antimicrobials. Local antimicrobial susceptibility is also reduced when biofilms form [36,37]. Because it is difficult to remove an established biofilm from an indwelling device, some experts advise removing catheters in cases of enterococcal CA-UTI and CA-BSI [38]. As a result, sanitation is important and neglecting it might attribute to clinical and microbiological failure.

This large, retrospective investigation found significant correlation between vancomycin AUC/MIC and clinical outcome and nephrotoxicity in the treatment of enterococcal infection. Before more randomized studies are carried out, our findings suggest that an AUC/MIC of ≥400 mg·h/L may be an ideal AUC/MIC for the application of the PK/PD parameters.

Because of the retrospective nature of our study, significant differences in the comparison groups between the therapy groups were observed, making comparisons difficult and contributing to uncertainty. However, we conducted a Cox regression analysis to assure that statistically significant clinical plausibility components were maintained in our final multi-variable model in order to address the most critical confounding factors. Secondly, the findings can only be generalized to university hospitals with similar patient populations and vancomycin sensitivities as ours because the study was limited to a single center. Thirdly, the use of clinical response as a primary endpoint for a cohort that has multiple other comorbid conditions which could also be responsible for the patient’s death. However, most retrospective research discovered this confounder. Although we used statistical approaches to control for potential confounders, this study design may still have residual undiscovered confounding factors. Fourthly, the findings of this study cannot be transferred to critically ill patients with remarkable changes in vancomycin PK. Furthermore, these findings are restricted to UTI and BSI. Concerning severity, enterococcal pneumonia and intraabdominal infections represent another class of infections.

## 5. Conclusions

This study reports a significant correlation between a vancomycin AUC/MIC of ≥400 mg·h/L and good clinical outcome in patients with *Enterococcus* infections and good microbiological outcome, when compared to a vancomycin AUC/MIC of <400 mg·h/L. However, a vancomycin AUC/MIC of ≥400 mg·h/L was associated with a high nephrotoxicity rate. Declining renal function may be a result of high vancomycin concentrations. In addition, declining renal function (e.g., failure to resolve the focus of infection, co-administration of other antibiotics) might result in higher AUC/MIC ratios. As a result of our findings, we suggest that the AUC_24_/MIC ratio of vancomycin ≥400 mg·h/L were recommended for the treatment of patients with enterococcal infection. In addition, patients with vancomycin AUC/MIC ≥400 mg·h/L were more likely to require aggressive, close monitoring of renal function. The findings are not transferable to critically ill patients with remarkable changes in vancomycin PK. Furthermore, these findings are restricted to UTI and BSI.

## Figures and Tables

**Table 1:** Baseline characteristics of patients with enterococcal infection treated with vancomycin (*n* = 312 cases).

Characteristic	AUC/MIC of <400 mg·h/L *n* = 78	AUC/MIC of ≥400 mg·h/L *n* = 234	*p*-Value
Male, n (%)	35 (44.87)	101 (43.16)	0.794
Female, n (%)	43 (55.13)	133 (56.84)	
Age, mean ± SD, years	60.32 ± 18.93	61.60 ± 17.91	0.637
Body weight, mean ± SD (kg)	56.06 ± 11.65	56.01 ± 12.55	0.975
Height, mean ± SD (cm)	165.8 ± 7.3	165.6 ± 7.1	0.830
Vancomycin dose, mean ± SD (mg/kg)	22.4 ± 5.2	28.4 ± 5.2	0.001
Diagnosis, n (%)			
Urinary tract infection	59 (75.64)	160 (68.38)	0.254
Bacteremia	8 (10.26)	28 (11.97)	0.838
Wound infection	6 (7.69)	33 (14.10)	0.168
Intra-abdominal infection	4 (5.13)	13 (5.56)	1.000
*Enterococcal* species, n (%)			
*E. faecium*	56 (71.79)	187 (79.91)	0.156
*E. feacalis*	22 (28.21)	47 (20.09)	
Comorbid disease states, n (%)			
Cardiovascular disease	28 (35.90)	79 (33.76)	0.783
Chronic kidney disease	22 (28.21)	90 (38.46)	0.133
Solid tumor	19 (24.36)	48 (20.51)	0.525
Diabetes mellitus	13 (16.67)	35 (14.96)	0.719
Cerebrovascular disease	7 (8.97)	15 (6.41)	0.448
Hematologic malignancy	1 (1.30)	7 (2.99)	0.684
Chronic liver disease	6 (7.69)	23 (9.87)	0.658
Respiratory disease	5 (6.41)	14 (5.98)	1.000
Others *	14 (17.95)	59 (25.21)	0.218
Co-contaminant nephrotoxic drugs, n (%)			
Furosemide	26 (33.33)	83 (35.47)	0.785
Piperacillin/tazobactam	10 (12.82)	34 (14.53)	0.851
Colistin	4 (5.13)	36 (15.38)	0.019
Aminoglycoside	2 (2.56)	2 (0.85)	0.261
Amphotericin B	5 (6.41)	5 (2.14)	0.128
Rifampin	1 (1.28)	5 (2.14)	1.000
Baseline Scr, mean ± SD, mg/dL	1.62 ± 1.91	2.10 ± 2.12	0.078
Baseline GFR, mean ± SD, ml/min	45.32 ± 37.48	36.59 ± 33.23	0.053
Duration of vancomycin therapy, mean ± SD, d	9.08 ± 4.98	10.50 ± 7.11	0.105
Duration of hospitalization, mean ± SD, d	34.43 ± 20.67	33.93 ± 21.85	0.858
APACHE II score, mean ± SD	12.42 ± 5.22	13.50 ± 5.52	0.130
Albumin	2.96 ± 0.65	2.91 ± 0.78	0.607
MIC of vancomycin for *Enterococcus* spp. mean ± SD, μg/mL	1.27 ± 0.71	0.90 ± 0.23	0.001
Vancomycin trough level, mean ± SD, mg/L	10.90 ± 4.61	23.42 ± 9.72	0.001

* other, gout, hyperthyroid, anemia; APACHE, acute physiology and chronic health evaluation; Scr, serum creatinine; SD, standard deviation.

**Table 2:** Pharmacokinetic parameters used for predictions **.

Parameter *	Mean	S.D.	Median	Min	Max
VS1 (L)	0.295907	0.195098	0.221463	0.044752	0.584645
KS1 (h^−1^)	0.004154	0.002980	0.003265	0.000843	0.009051
KPC (h^−1^)	0.556593	0.699659	0.392091	0.080472	3.047390
KCP (h^−1^)	1.366962	1.897641	0.898388	0.107569	7.998810
KI (h^−1^)	0.002043	0	0.002043	0.002043	0.002043

* KCP = rate constant from central to peripheral compartment; KI = rate constant of nonrenal elimination; KPC = rate constant from peripheral to central compartment; KS1 = rate constant of renal elimination, such that coefficient of elimination = KS1 × GFR + KI; VS1 = volume of distribution of central compartment. ** Population parameters derived from BestDose software.

**Table 3:** Cox regression analysis of outcomes for enterococcal infection patients with vancomycin AUC/MIC of ≥400 mg·h/L.

Variable	AUC/MIC of <400 mg·h/L, n (%)	AUC/MIC of ≥400 mg·h/L, n (%)	Crude HR (95% CI)	*p*-Value	Adjusted HR * (95% CI)	*p*-Value
**Efficacy**						
Clinical failure	17 (22.97)	37 (15.55)	0.59 (0.32–1.04)	0.072	0.50 (0.26–0.97)	0.042
Microbiological failure	9 (12.16)	16 (6.72)	0.44 (0.20–0.99)	0.050	0.37 (0.14–0.94)	0.036
**Safety**						
Nephrotoxicity	3 (3.85)	35 (14.96)	3.34 (1.02–10.91)	0.046	3.96 (1.09–14.47)	0.037

HR, hazard ratio; CI, confidence interval; * Adjusted for gender, APACHE II score, albumin, species, CKD, baseline Scr, Amphotericin B, MIC, Aminoglycoside.

## Data Availability

The datasets used and analyzed during the current study are available from the corresponding author on reasonable request.

## References

[1] Schröder U.C., Beleites C., Assmann C., Glaser U., Hübner U., Pfister W., Fritzsche W., Popp J., Neugebauer U. (2015). Detection of vancomycin resistances in enterococci within 3½ hours. Sci. Rep..

[2] NARST Percent Susceptibility of *Enterococci*. http://narst.dmsc.moph.go.th/data/AMR%202000-2020-06M.pdf.

[3] Deryke C.A., Alexander D.P. (2009). Optimizing vancomycin dosing through pharmacodynamic assessment targeting area under the concentration-time curve/minimum inhibitory concentration. Hosp. Pharm..

[4] Rybak M., Lomaestro B., Rotschafer J.C., Moellering R., Craig W., Billeter M., Dalovisio J.R., Levine D.P. (2009). Therapeutic monitoring of vancomycin in adult patients: A consensus review of the American society of health-system pharmacists, the infectious diseases society of America, and the society of infectious diseases pharmacists. Am. J. Health-Syst. Pharm..

[5] Moise-Broder P.A., Forrest A., Birmingham M.C., Schentag J.J. (2004). Pharmacodynamics of vancomycin and other antimicrobials in patients with Staphylococcus aureus lower respiratory tract infections. Clin. Pharm..

[6] Kullar R., Davis S.L., Levine D.P., Rybak M.J. (2011). Impact of vancomycin exposure on outcomes in patients with methicillin-resistant *Staphylococcus aureus* bacteremia: Support for consensus guidelines suggested targets. Clin. Infect. Dis..

[7] Matsumoto K., Takesue Y., Ohmagari N., Mochizuki T., Mikamo H., Seki M., Takakura S., Tokimatsu I., Takahashi Y., Kasahara K. (2013). Practice guidelines for therapeutic drug monitoring of vancomycin: A consensus review of the Japanese society of chemotherapy and the Japanese society of therapeutic drug monitoring. J. Infect. Chemother..

[8] Rybak M.J., Le J., Lodise T.P., Levine D.P., Bradley J.S., Liu C., Mueller B.A., Pai M.P., Wong-Beringer A., Rotschafer J.C. (2020). Therapeutic monitoring of vancomycin for serious methicillin-resistant Staphylococcus aureus infections: A revised consensus guideline and review by the American society of health-system pharmacists, the infectious diseases society of america, the pediatric infectious diseases society, and the society of infectious diseases pharmacists. Am. J. Health-Syst. Pharm..

[9] Jumah M., Vasoo S., Menon S.R., De P.P., Neely M., Teng C.B. (2018). Pharmacokinetic/pharmacodynamic determinants of vancomycin efficacy in enterococcal bacteremia. Antimicrob. Agents Chemother..

[10] Nakakura I., Sakakura K., Imanishi K., Sako R., Yamazaki K. (2019). Association between vancomycin pharmacokinetic/pharmacodynamic parameters, patient characteristics, and mortality in patients with bacteremia caused by vancomycin-susceptible *Enterococcus faecium*: A single-center retrospective study. J. Pharm. Health Care Sci..

[11] Gawronski K.M., Goff D.A., Brown J., Khadem T.M., Bauer K.A. (2013). A stewardship program’s retrospective evaluation of vancomycin AUC24/MIC and time to microbiological clearance in patients with methicillin-resistant *Staphylococcus aureus* bacteremia and osteomyelitis. Clin. Ther..

[12] Suzuki Y., Kawasaki K., Sato Y., Tokimatsu I., Itoh H., Hiramatsu K., Takeya-ma M., Kadota J. (2012). Is peak concentration needed in therapeutic drug monitoring of vancomycin? A pharmacokinetic-pharmacodynamic analysis in patients with methicillin-resistant staphylococcus aureus pneumonia. Chemotherapy.

[13] Horan T.C., Andrus M., Dudeck M.A. (2008). CDC/NHSN surveillance definition of health care-associated infection and criteria for specific types of infections in the acute care setting. Am. J. Infect. Control..

[14] Roustit M., François P., Sellier E., Roch N., Vittoz J.P., Foroni L., Stahl J.P., Pavese P. (2010). Evaluation of glycopeptide prescription and therapeutic drug monitoring at a university hospital. Scand. J. Infect. Dis..

[15] Jelliffe R.W., Bayard D., Schumitzky A., Milman M., Van Guilder M. (1995). Pharmaco-Informatics: More precise drug therapy from ‘multiple model’ (MM) adaptive control regimens: Evaluation with simulated vancomycin therapy. Medinfo.

[16] Nunn M.O., Corallo C.E., Aubron C., Poole S., Dooley M.J., Cheng A.C. (2011). Vancomycin dosing: Assessment of time to thera-peutic concentration and predictive accuracy of pharmacokinetic modeling software. Ann. Pharmacother..

[17] Felton T.W., Roberts J.A., Lodise T.P., Van Guilder M., Boselli E., Neely M.N., Hope W.W. (2014). Individualization of piperacillin dosing for critically ill patients: Dosing software to optimize antimicrobial therapy. Antimicrob. Agents Chemother..

[18] Marsot A., Boulamery A., Bruguerolle B., Simon N. (2012). Vancomycin: A review of population pharmacokinetic analyses. Clin. Pharm..

[19] Lodise T.P., Drusano G.L., Zasowski E., Dihmess A., Lazariu V., Cosler L., McNutt L.A. (2014). Vancomycin exposure in patients with methicillin-resistant *Staphylococcus aureus* bloodstream infections: How much is enough?. Clin. Infect. Dis..

[20] CLSI (2015). Performance Standards for Antimicrobial Susceptibility Testing.

[21] Levison M.E., Levison J.H. (2009). Pharmacokinetics and pharmacodynamics of antibacterial agents. Infect. Dis. Clin. N. Am..

[22] Hanberger H., Nilsson L.E., Maller R., Isaksson B. (1991). Pharmacodynamics of daptomycin and vancomycin on *Enterococcus faecalis* and *Staphylococcus aureus* demonstrated by studies of initial killing and postantibiotic effect and influence of Ca^2+^ and albumin on these drugs. Antimicrob. Agents Chemother..

[23] Lodise T.P., Lomaestro B., Graves J., Drusano G.L. (2008). Larger vancomycin doses (at least four grams per day) are associated with an increased incidence of nephrotoxicity. Antimicrob. Agents Chemother..

[24] Neely M.N., Kato L., Youn G., Kraler L., Bayard D., van Guilder M., Schumitzky A., Yamada W., Jones B., Minejima E. (2018). Prospective trial on the use of trough concentration versus area under the curve to determine therapeutic vancomycin dosing. Antimicrob. Agents Chemother..

[25] Muklewicz J.D., Steuber T.D., Edwards J.D. (2021). Evaluation of area under the concentration-time curve-guided vancomycin dosing with or without piperacillin-tazobactam on the incidence of acute kidney injury. Int. J. Antimicrob. Agents..

[26] Zasowski E.J., Murray K.P., Trinh T.D., Finch N.A., Pogue J.M., Mynatt R.P., Rybak M.J. (2017). Identification of vancomycin exposure-toxicity thresholds in hospitalized patients receiving intravenous vancomycin. Antimicrob. Agents Chemother..

[27] Cristallini S., Hites M., Kabtouri H., Roberts J.A., Beumier M., Cotton F., Lipman J., Jacobs F., Vincent J.L., Creteur J. (2016). New regimen for continuous infusion of vancomycin in critically Ill patients. Antimicrob. Agents Chemother..

[28] Hanrahan T.P., Harlow G., Hutchinson J., Dulhunty J.M., Lipman J., Whitehouse T., Roberts J.A. (2014). Vancomycin-Associated nephrotoxicity in the critically ill: A retrospective multivariate regression analysis. Crit. Care Med..

[29] Hanrahan T., Whitehouse T., Lipman J., Roberts J.A. (2015). Vancomycin-Associated nephrotoxicity: A meta-analysis of administration by continuous versus intermittent infusion. Int. J. Antimicrob. Agents..

[30] Katip W., Jaruratanasirikul S., Pattharachayakul S., Wongpoowarak W., Jitsurong A., Lucksiri A. (2016). The pharmacokinetics of vancomycin during the initial loading dose in patients with septic shock. Infect. Drug Resist..

[31] Flores-Mireles A.L., Walker J.N., Potretzke A., Schreiber H.L., Pinkner J.S., Bauman T.M., Park A.M., Desai A., Hultgren S.J., Caparon M.G. (2016). Antibody-Based therapy for enterococcal catheter-associated urinary tract infections. mBio.

[32] Marschall J., Piccirillo M.L., Fraser V.J., Doherty J.A., Warren D.K. (2013). Catheter removal versus retention in the management of catheter-associated enterococcal bloodstream infections. Can. J. Infect. Dis. Med. Microbiol..

[33] Assadi F. (2018). Strategies for preventing catheter-associated urinary tract infections. Int. J. Prev. Med..

[34] Sandoe J., Witherden I.R., Cove J.H., Heritage J., Wilcox M.H. (2003). Correlation between enterococcal biofilm formation In Vitro and medical-device-related infection potential In Vivo. J. Med. Microbiol..

[35] Raad I.I., Hanna H.A., Boktour M., Chaiban G., Hachem R.Y., Dvorak T., Lewis R., Murray B.E. (2005). Vancomycin-Resistant *Enterococcus faecium*: Catheter colonization, esp gene, and decreased susceptibility to antibiotics in biofilm. Antimicrob. Agents Chemother..

[36] Sandoe J.A., Wysome J., West A.P., Heritage J., Wilcox M.H. (2006). Measurement of ampicillin, vancomycin, linezolid and gentamicin activity against enterococcal biofilms. J. Antimicrob. Chemother..

[37] Wiederhold N.P., Coyle E.A., Raad I.I., Prince R.A., Lewis R.E. (2005). Antibacterial activity of linezolid and vancomycin in an in vitro pharmacodynamic model of Gram-positive catheter-related bacteraemia. J. Antimicrob. Chemother..

[38] Mermel L.A., Allon M., Bouza E., Craven D.E., Flynn P., O’Grady N.P., Raad I.I., Rijnders B.J., Sherertz R.J., Warren D.K. (2009). Clinical practice guidelines for the diagnosis and management of intravascular catheter-related infection: 2009 update by the infectious diseases society of America. Clin. Infect. Dis..

